# The role of saline irrigation prior to wound closure in the reduction of surgical site infection: protocol for a systematic review and meta-analysis

**DOI:** 10.1186/s13643-018-0813-7

**Published:** 2018-10-05

**Authors:** Dawid Pieper, Tanja Rombey, Johannes Doerner, Julian-Dario Rembe, Hubert Zirngibl, Konstantinos Zarras, Peter C. Ambe

**Affiliations:** 1 Institute for Research in Operative Medicine, Chair of Surgical Research, Faculty of Health, School of Medicine, Witten/Herdecke University, Witten, Germany; 2 Department of Surgery, Helios University Hospital Wuppertal, Witten/Herdecke University, Heusnerstr. 40, 42283 Wuppertal, Germany; 3 Department of visceral, minimal invasive and oncologic surgery, Marienhospital Düsseldorf, Rochusstr. 2, 40479 Düsseldorf, Germany

## Abstract

**Background:**

Surgical site infection describes an infectious complication of surgical wounds. This single complication is thought to occur in close to 20% of surgical cases. This complication has been described in all kinds of surgical procedure including minimally invasive procedures. Wound irrigation is frequently used as a means of reducing surgical site infection. However, there is lack of solid evidence to support routine wound irrigation. The aim of this review is to provide evidence for the efficacy of routine wound irrigation with normal saline in preventing surgical site infection. The rate of surgical site infection in cases with and without wound irrigation will be analyzed.

**Methods/design:**

Systematic literature searches will be conducted to identify all published and unpublished studies. The following databases will be searched for citations from inception to present: MEDLINE (via PubMed), Embase (via Embase), and CENTRAL (via the Cochrane library). The search strategy will be developed by the research team in collaboration with an experienced librarian and checked by a referee according to the Peer Review of Electronic Search Strategies (PRESS) guideline. A draft of the PubMed search strategy could be (irrigation[tiab] OR “Therapeutic Irrigation”[mesh] OR lavage[tiab]) AND (saline[tiab] OR “Sodium Chloride”[mesh] OR sodium chloride[tiab]) NOT (“Comment” [Publication Type] OR “Letter” [Publication Type] OR “Editorial” [Publication Type]). No time limits will be set. The reference lists of eligible articles will be hand searched. Relevant data will be extracted from eligible studies using a previously designed data extraction sheet. Relative risks will be calculated for binary outcomes and mean differences or standardized mean differences, if necessary, for continuous outcomes. For all measures, 95% confidence levels will be calculated. Both arms would be compared with regard to the rate of surgical site infection within 30 days following surgery. We will report the review using the Preferred Reporting Items for Systematic Reviews and Meta-Analysis (PRISMA) statement.

**Discussion:**

This review aims at investigating the value of routine wound irrigation using normal saline in preventing surgical site infection.

**Systematic review registration:**

PROSPERO: CRD42018082287

## Introduction

### Rationale

According to the World Health Organization (WHO), healthcare-associated infections (HAIs) acquired by patients when receiving care is the most frequent adverse event worldwide. Urinary tract infection, pneumonia, bloodstream, and wound infections with microorganisms represent the most common HAIs [1]. Surgical site infection (SSI) describes an infectious complication of surgical wounds. With an incidence of about 20%, SSI has been shown to be the most common HAI [2]. SSI surveillance report from the European Centre for Disease Prevention and Control for 2011 indicated a cumulative SSI incidence of 9.5% for colorectal surgery, 1.4% for cesarean section, and 1.0% for cholecystectomy [3].

SSI is a potential complication associated with any type of surgery irrespective of access (minimal invasive or open) or surgical discipline. Although SSI is thought to be preventable, it still represents a major cause of morbidity and substantial economic burden on the health system [4]. Many study groups and international guidelines have suggested measures to prevent or at least reduce the rate of SSI [1, 5–8]. The use of prophylactic single-shot antibiotics at the beginning of surgery prior to skin incision, minimally invasive access with less tissue trauma, and the use of wound protectors have been described as means of reducing SSI [5, 9–11].

Wound irrigation (WI) describes the flow of a solution across the surface of a surgical incision prior to wound closure [12]. Wound irrigation is intended to cleanse the wound physically by removing cellular debris and trapped fluids. Wound irrigation might reduce the level of bacterial contamination by flushing off bacteria from the wound surface [13].

Different forms of WI have been described in the medical literature. WI can be achieved using normal saline, antibiotic solutions, and solutions with antiseptic agents [14–16]. Despite the large number of publications on WI, no single method or solution has been shown to be superior. Besides, there seems to be conflicting data on the role of WI on SSI [17, 18]. In fact, some guidelines do not recommend routine WI [5, 7]. This trend can be observed across different surgical disciplines [19, 20].

In our department of surgery at a university hospital, WI using normal saline is routinely performed and the rate of SSI is very low. Current data on the role of WI as a means of reducing or preventing SSI is conflicting. Based on our clinical experience, we hypothesize that WI with normal saline might reduce the rate of SSI. However, no systematic review to the best of our knowledge has so far investigated WI with normal saline alone. We therefore intend to investigate the potential role of WI with normal saline in preventing SSI by performing a systematic review of randomized controlled trials (RCTs).

### Objectives

We aim to investigate the efficacy of WI with normal saline in reducing surgical site infection. The rate of SSI in patients following wound irrigation with normal saline will be compared to that of patients without wound irrigation prior to wound closure.

## Methods

This protocol adheres to the Preferred Reporting Items for Systematic Review and Meta-Analysis-Protocols (PRISMA-P) [21]. Any amendments to this protocol will be reflected in an update to the PROSPERO registration.

### Eligibility criteria

RCTs investigating the rate of SSI in using normal saline vs. no irrigation prior to wound closure following abdominal surgery will be included. Only studies published in English and German language will be included. Relevant studies in languages other than English will be considered if an English translation is available. In such cases, a translation will be requested from the corresponding authors. All available publications irrespective of date of publication will be considered.

#### Population

All patients undergoing abdominal surgery irrespective of diagnosis, procedure, and discipline will be considered for inclusion. Patients undergoing minimally invasive surgery will be included if a mini-laparotomy of more than 3 cm was performed, e.g., for specimen retrieval. Patients undergoing natural orifice procedures (NOTES) will not be included. Patients undergoing both elective and emergency surgery will be included. No differences will be made between patients with or without drains. The same role holds for the use of wound protectors.

#### Intervention

The study group will include all patients who underwent saline irrigation of the surgical incision prior to wound closure. All forms of saline wound irrigation with or without the use of a pressure device or syringe will be included in this intervention arm. The control group will include patients without irrigation.

#### Comparison

All comparisons will be considered.

#### Outcome

Primary outcome will be SSI. As secondary outcomes, we will consider length of stay (LOS), rate of re-intervention, rate of re-admission, overall morbidity, mortality, quality of life, and the resource use for treatment.

#### Study designs

We will only include (quasi-) RCTs in our systematic review. Systematic reviews related to the topic will be retained to investigate their references for further eligible studies.

### Information sources

We will conduct a systematic literature search to identify all published and unpublished studies. The following databases will be searched for citations from inception to present: MEDLINE (via PubMed), Embase (via Embase), and CENTRAL (via the Cochrane library). We will search manually for additional studies by cross-checking the reference lists of all included primary studies and lists of relevant systematic reviews.

We will not apply any limitations regarding language, publication status, and publication date when searching for eligible studies.

### Search strategy

The search strategy will be developed by the research team in collaboration with an experienced librarian and checked by a referee according to the Peer Review of Electronic Search Strategies (PRESS) guideline. A draft of the PubMed search strategy can be found below:

(irrigation[tiab] OR “Therapeutic Irrigation”[mesh] OR lavage[tiab]) AND (saline[tiab] OR “Sodium Chloride”[mesh] OR sodium chloride[tiab])

AND

((randomized controlled trial[pt] OR controlled clinical trial[pt] OR randomized[tiab] OR randomised[tiab] OR placebo[tiab] OR clinical trials as topic[mesh:noexp] OR randomly[tiab] OR trial[ti] NOT (animals[mh] NOT humans [mh]))) NOT (“Comment” [Publication Type] OR “Letter” [Publication Type] OR “Editorial” [Publication Type])

### Data management

The search results will be uploaded and managed using Microsoft Excel.

### Selection process

The title and abstract of each article will be screened and assessed against predetermined inclusion criteria by two independent investigators. Full texts of all potentially relevant articles and those without an available abstract will be assessed for inclusion by two reviewers independently. Discrepancies will be resolved by consensus or consulting a third investigator. The corresponding authors of eligible articles will be contacted for clarification where necessary. We will record the reasons for exclusion and report the study selection process using the PRISMA flow diagram [22]. A list of excluded studies will be provided.

### Data collection process

A data extraction sheet will be designed and tested. Two reviewers will independently extract data from the included studies. Any disagreements will be resolved via discussion or by involving a third reviewer for arbitration.

### Data items

We will collect data on patient’s demographics (age, sex, body mass index), relevant medical conditions (ASA score, diabetes, immune status), type of surgical procedure and means of access (laparoscopic or open), information on lifestyle, e.g., smoking, and perioperative data including procedure-associated information like type of surgery, duration of surgery, use of single-shot antibiotics, and use of wound drains. Furthermore, we will extract the number of randomized patients included in the analysis.

The primary outcome will be the number of SSI. Secondary outcomes will include mean or median LOS, number of re-intervention, specific medical treatment for SSI (e.g., antibiotics), number of readmission, overall morbidity, number of deaths, and resource use (e.g., hospital length of stay, costs). In case outcome data are missing, we will contact study authors and request the data.

### Risk of bias in individual studies

We will use the revised Cochrane Risk of Bias tool to evaluate all included studies for risk of bias [23]. Items will be rated as low, high, or unclear risk of bias. Independently, two reviewers will assess the risk of bias of all included studies. We will assess the risk of bias on outcome level. For each assessment, we will provide a support for judgment. Any disagreements will be resolved through discussion. If both reviewers cannot find a consensus, we will involve a third reviewer.

### Summary measures and synthesis

Relative risks will be calculated for binary outcomes and mean differences or standardized mean differences, if necessary, for continuous outcomes. For all measures, 95% confidence levels will be calculated.

Clinical and statistical heterogeneity between studies will be assessed by two reviewers. For the assessment of statistical heterogeneity, *I*^2^ will be calculated. In the absence of clinical heterogeneity, and in the presence of statistical heterogeneity (*I*^2^ > 50%), we will use a random-effects model. In case of no clinical or statistical heterogeneity, we will apply a fixed-effect model. We will obtain pooled estimates of treatment effect using RevMan 5 software.

We do not plan any additional analyses, such as subgroup or sensitivity analyses. However, we may decide to perform post hoc analyses given appropriate numbers.

### Meta-bias

If there are 10 or more studies included in the meta-analysis, we will investigate publication bias using funnel plots and Egger’s test [24].

### Confidence in cumulative evidence

Summary of finding tables will be prepared for summarizing confidence across studies for all patient relevant outcomes. For grading the quality of evidence, the five GRADE domains, risk of bias, indirectness, inconsistency, imprecision, and publication bias, will be judged. The quality of the body of evidence will be assessed by two reviewers independently using the GRADEpro GDT software.

## Discussion

Two out of ten patients undergoing abdominal surgery will suffer from SSI, which causes an increase in overall morbidity rate [2]. Besides, SSI might lead to prolongation of LOS [25, 26]. The economic burden imposed on the health system by SSI cannot be overemphasized. Although many institutions implemented measures to reduce the risk of SSI, this single complication is still a cause of serious morbidity and expenses [27].

Although wound irrigation has been widely investigated in association with surgical site infection, consistent data supporting the benefit of WI is lacking. One reason for this trend might be the heterogeneity of the study population with regard to wound irrigation. Antibiotic solution, solution with antiseptic agents, normal saline, and other combinations have so far been used for wound irrigation. This observation was confirmed in a recently published systematic review by Mueller et al. [28].

Most solutions used for irrigation except saline are not inert. It is thus possible that substances in the irrigation solution might negatively affect wound healing thereby predisposing to SSI. We routinely perform wound irrigation using saline in our department of abdominal surgery. Our clinical experience suggests a potential reduction in the rate of SSI following routine WI with saline. Currently, there is limited evidence on the role of normal saline irrigation on the rate of SSI.

This systematic review will compute data from RCTs to clarify the role of routine WI with normal saline in the reduction or prevention of SSI. The risk of SSI in patients managed with normal saline irrigation will be compared to that of controls without WI. Secondary endpoints like the rate of morbidity, length of stay, and overall resource use of treatment will be investigated.

The results generated in the planned systematic review might provide solid evidence to rethink our routine clinical processes or adjust the current recommendations for the prevention of surgical site infection. It is also conceivable that the planned systematic review might not indicate any trend, which would reveal the need for further investigation, e.g., via additional RCTs.
